# Long noncoding RNA SNHG17 induced by YY1 facilitates the glioma progression through targeting miR-506-3p/CTNNB1 axis to activate Wnt/β-catenin signaling pathway

**DOI:** 10.1186/s12935-019-1088-3

**Published:** 2020-01-28

**Authors:** Huixia Li, Tianhao Li, Dehai Huang, Peng Zhang

**Affiliations:** 1 Reception Office, The First Affiliated Hospital of Zhengzhou University, Zhengzhou, 450000 Henan China; 2 Department of Pathogen Biology and Immunology, Henan Medical College, Zhengzhou, 450000 Henan China; 3 The First Affiliated Hospital of Zhengzhou University, No. 186 Worker Road, Zhongyuan District, Zhengzhou, Henan China

**Keywords:** SNHG17, YY1, miR-506-3p, CTNNB1, Glioma

## Abstract

**Background:**

Glioma is one of the most widely diagnosed malignancies worldwide. It has been reported that long noncoding RNAs (lncRNAs) are participators in the tumorgenesis of cancers. Nevertheless, the role and function of lncRNA SNHG17 among glioma is unclear.

**Methods:**

RT-qPCR revealed SNHG17, YY1, miR-506-3p, CTNNB1 expression among glioma cells. CCK-8, colony formation, EdU, flow cytometry, TUNEL and western blot assays revealed the function of SNHG17 in glioma. RIP uncovered SNHG17, miR-506-3p and CTNNB1 enrichment in RISC complex. Luciferase reporter assays and RNA pull down revealed interaction of miR-506-3p with SNHG17 and CTNNB1.

**Results:**

SNHG17 expression was up-regulated in glioma tissues and cells. SNHG17 silence attenuated cell proliferation and promoted apoptosis and repressed tumor growth. Moreover, SNHG17 was up-regulated by transcription factor YY1. Mechanistically, SNHG17 activated Wnt/β-catenin signaling pathway in glioma. CTNNB1 was referred to as the mRNA of β-catenin, we validated that SNHG17 bound to miR-506-3p to induce CTNNB1 and activate Wnt/β-catenin signaling pathway. Rescue experiments indicated that CTNNB1 overexpression abolished the inhibitory effects of SNHG7 inhibition on glioma progression.

**Conclusions:**

The findings that YY1-induced SNHG17 facilitated the glioma progression through targeting miR-506-3p/CTNNB1 axis to activate Wnt/β-catenin signaling pathway offered a brand-new prospects to molecular-targeted treatment for glioma.

## Background

Glioma is publicly received as one common primary tumor in central nervous system featured by high recurrence along with mortality rate [1]. Glioma includes astrocytoma, oligodendroglioma, ependymoma and mixed tumor according to histological subtypes and malignant degree [2]. Present therapeutic methods for glioma are surgery, chemotherapy and radiotherapy [3, 4]. Although great improvements have been achieved over the last years, it was sad to see that the overall survival rate of most glioma patients is still dismal which results in a situation where glioma is a main contributing factor of cancer-associated death worldwide [5, 6]. Therefore, it is essential to explore effective strategies which can reduce the incidence and mortality of glioma to improve the results of glioma therapy.

Long noncoding RNAs (lncRNAs) are a class of non-coding RNAs whose length is more than 200 nucleotides [7]. LncRNAs can modulate gene expression through multiple mechanisms, such as controlling of transcription, posttranscriptional, genomic imprinting, modification of chromatin and regulating the function of the protein [8]. Thus, lncRNAs exert pivotal part in different biological processes [9, 10]. The discovery of lncRNA has provided a novel investigating target to uncover the therapeutic methods for human diseases. Till now, many studies have proved the correlation between lncRNAs and cancer pathogenesis. For instance, LncRNA MALAT1 exerts critical function on metastasis in lung cancer [11]. LncRNA SCAMP1 facilitates human pancreatic and gallbladder cancer cell migration and invasion [12]. LncRNA SNHG17 was confirmed to be involved in the progression of several cancers. For example, LncRNA SNHG17 aggravated cell proliferation, and migration as along with reduces cell apoptosis via down-regulation of p15 and p57 in gastric cancer [13]. LncRNA SNHG17 modulated human NSCLC cell proliferation and migration [14]. However, current studies about lncRNAs are limited, and the role and deep-going regulatory mechanism of lncRNA SNHG17 in glioma remain to be elucidated.

Wnt//β-catenin signaling pathway is validated to exert tremendous effects on the development of various cancers. The function of Wnt signaling pathway counts on β-catenin, which is the key part in this signaling. For instance, LINC00210 activated Wnt//β-catenin activity and contributed to process of liver tumor by targeting CTNNB1P1 [15].In this study, we found that LiCl could rescue the impacts of SNHG17 on the course of glioma and then we delved into how SNHG17 had impacts on Wnt signaling pathway.

In our research, we aimed at uncovering the role and deep-going regulatory mechanism of lncRNA SNHG17 in glioma. The results proved that SNHG17 induced by YY1 facilitated the glioma progression through targeting miR-506-3p/CTNNB1 axis by activation of Wnt/β-catenin signaling pathway, suggesting its potential value as a biomarker in glioma.

## Materials and methods

### Tissue samples

33 matched samples of glioma tissues and adjacent normal tissues were attained from the First Affiliated Hospital of Zhengzhou University. This study got approval from the ethics committee of the First Affiliated Hospital of Zhengzhou University. In this study, all participants signed informed consent forms and no patients had received chemotherapy or radiotherapy before surgery. Tissue specimens were stored at − 80 °C right after surgical resection for further analysis.

### Cell culture and treatment

Normal human astrocyte cell (NHA) and human glioma cells (U87, U251, SHG44 and A172) were attained from American Type Culture Collection (ATCC; Manassas, VA, USA). In a humidified atmosphere with 5% CO_2_, cells were propagated with Dulbecco’s Modified Eagle Medium (DMEM; Gibco, Rockville, MD, USA) with additional 10% FBS (Gibco) and 1% penicillin/streptavidin (Gibco) at 37 °C. SC79, CD40L, LiCl, Jagged1 and IWR-1-endo were acquired from Sigma-Aldrich (St. Louis, MO, USA) to treat cells, individually. IWR-1-endo was one of the inhibitors of Wnt response.

### Cell transfection

Specific shRNAs against SNHG17 (sh-SNHG17#1, sh-SNHG17#2 and sh-SNHG17#3) or YY1 (sh-YY1) and their corresponding NCs (sh-NCs), together with the pcDNA3.1 vector targeting SNHG17 or YY1 or CTNNB1 and the empty vector, were constructed by Genechem (Shanghai, China). Moreover, miR-506-3p mimics and NC mimics were designed by GenePharma (Shanghai, China). U87 or U251 cells were transfected with these plasmids via Lipofectamine 3000 (Invitrogen, Carlsbad, CA, USA), severally.

### RT-qPCR

Total RNA was extracted utilizing TRIzol reagent (Sigma-Aldrich). Complementary DNA (cDNA) was sequentially synthesized by M-MLV reverse transcriptase (Thermo Fisher Scientific, Waltham, MA, USA). RT-qPCR was implemented on ABI Prism 7900HT (Applied Biosystems, Foster City, CA, USA) applying SYBR GREEN PCR Master (Applied Biosystems). To detect miRNA, we applied stem-loop method to prolong the RNA into a detectable length (80–100 nt) and the stem-loop specific primer and the reverse complementary sequence were applied as two primers for RT-qPCR. All data were normalized to GAPDH or U6 based on 2^−ΔΔCt^ method.

### Cell proliferation assay

Cell Counting Kit 8 (CCK-8) solution (Dojindo, Osaka, Japan) was acquired for undertaking cell proliferation assay. Transfected U87 or U251 cells with or without treatment were fixed in 96-well plates. Cell proliferation was observed at indicated time points via measuring absorbance at 450 nm with a spectrophotometer (Thermo Fisher Scientific). Prior to measurement, each well was supplemented with CCK-8 reagent and cultured for another 3 h.

### Colony formation assay

Transfected U87 or U251 cells with or without treatment were added into 6-well plates. Following culture for 14 days, the resulting colonies were fixed for 30 min in formaldehyde (Sigma-Aldrich) and sequentially stained for 5 min by using crystal violet (Sigma-Aldrich). Colonies were photographed and counted.

### EdU staining

EdU staining was implemented with a BeyoClick™ EdU Cell Proliferation Kit with Alexa Fluor 594 (Beyotime, Shanghai, China). Transfected U87 or U251 cells with or without treatment were rinsed with PBS (Sigma-Aldrich). Upon addition of fresh medium, EdU was added and cells were incubated for 2 h. After incubation, cells were washed by PBS so as to remove the medium as well as the free EdU probe. Next, cells were immobilized for 30 min in PFA (Sigma-Aldrich) before being stained by DAPI (Sigma-Aldrich). Following an additional wash with PBS, cells were observed via an inverted microscope (Olympus, Tokyo, Japan).

### Flow cytometry

Cell apoptosis of transfected U87 or U251 cells with or without treatment were studied with a flow cytometer (BD Biosciences, Franklin Lakes, NJ, USA) plus double Annexin V/PI staining (Invitrogen). Simply, transfected U87 or U251 cells with or without treatment were harvested from 6-well plates through centrifugation and mixed with 1X binding buffer (Invitrogen), after which were dyed by FITC-Annexin V and propidium iodide (PI). Apoptotic cells were assayed by flow cytometry.

### TUNEL assay

Transfected U87 or U251 cells with or without treatment were washed utilizing PBS and fixed by PFA. TUNEL reagents (Merck KGaA, Darmstadt, Germany) were applied in order to stain apoptotic cells. The optical microscopy (Olympus) was acquired for analysis.

### Western blot

Equal amounts of protein were applied to 10% SDS-PAGE (Bio-Rad, Hercules, CA, USA) and subsequently transferred to PVDF membranes (Bio-Rad). Membranes were blocked by 5% non-fat dry milk in Tris Buffered Saline with Tween^®^ 20 (TBS-T; Sigma-Aldrich) and then incubated at 4 °C with primary antibodies overnight to reveal the expression of them. Next, membranes were washed with TBS-T, followed by incubated for 1 h with specific secondary antibodies. Peroxidase activity were visualized with the enhanced chemiluminescence substrate system (ECL; Santa Cruz Biotechnology, Santa Cruz, CA, United States) and the bands were quantified using Quantity One (Bio-Rad). Primary antibodies against cleaved-caspase3 (ab32042, Abcam, Cambridge, USA), total caspase3 (ab32351, Abcam), cleaved-caspase9 (ab2324, Abcam), total caspase9 (ab32539, Abcam), Bax (ab32503, Abcam) β-catenin (ab32572, Abcam) and GAPDH (ab245356, Abcam) were employed, separately.

### Chromatin immunoprecipitation (ChIP)

ChIP assay was implemented by use of the EZ ChIP™ Chromatin Immunoprecipitation Kit for cell line (Millipore, Bedford, MA). U87 or U251 cell samples were initially fixed in the 1% PFA for 15 min’s crosslink then processed by ultrasonic for cutting DNA into fragments (500 bp). Following that, the 6 h of immunoprecipitation was undertaken with antibodies against control IgG and YY1, respectively. 30 μl of magnetic beads were added afterwards to collect the precipitated chromatin for RT-qPCR analysis.

### Luciferase reporter assay

SNHG17 promoter-WT/Mut was inserted into the pGL3-basic vector (Promega, Madison, WI, USA) to form pGL3-SNHG17 promoter-WT/Mut which was then co-transfected with pcDNA3.1/YY1 or the empty vector into U87 or U251 cells. The 3′ untranslated region (3′UTR) of CTNNB1 which contained the miR-506-3p binding site (UGCCUU) was inserted into pmirGLO as CTNNB1-WT the mutant type which could not bind to miR-506-3p was called CTNNB1-Mut. CTNNB1-WT/Mut was inserted into the pmirGLO dual-luciferase plasmid (Promega) to form pmirGLO-CTNNB1-WT/Mut which was co-transfected with miR-506-3p mimics or miR-506-3p mimics + pcDNA3.1/SNHG17 or NC mimics into U87 or U251 cells. Relative luciferase activities were studied using dual-luciferase reporter assay system (Promega).

### TOP/FOP assay

U87 or U251 cells were co-transfected with sh-SNHG17#1 or sh-NC and TOP Flash or FOP Flash (Upstate Biotechnology, Lake Placid, NY, USA). Relative luciferase activities were studied using dual-luciferase reporter assay system (Promega).

### Pulldown assay

RNA pulldown assay were implemented as formerly described [16]. Briefly, miR-506-3p and miR-506-3p (Mut) were biotin-labeled using Biotin RNA Labeling Mix (Roche) and transcribed with T7/SP6 RN polymerase (Roche Diagnostics, Indianapolis, IN, USA). Thereafter, the bio-miR-506-3p-WT, bio-miR-506-3p-Mut, or control miR-NC were incubated with lysates of the U87 and U251 cells, mixed with the M-280 streptavidin magnetic beads (Invitrogen, San Diego, CA, USA). Following the washing, the RNAs pulled down were evaluated applying qRT-PCR.

### RNA immunoprecipitation (RIP)

Applying a Magna RIP™ RNA-Binding Protein Immunoprecipitation Kit (Millipore), RIP was undertaken with anti-Ago2 antibody or anti-IgG attained from Abcam. The cultured U87 or U251 cell lines in PBS were first lysed in RIP lysis buffer then collected. Cell lysates were processed with the RIP buffer adding the antibody-bound magnetic beads. Next, samples were digested, immunoprecipitated RNAs were extracted and purified. RT-qPCR was performed to confirm the presence of the binding sites.

### Xenograft model

BALB/c nude mice (3 weeks old) were purchased from Slac Laboratories (Shanghai, China). Glioma cells transfected with sh-SNHG17#1 or sh-NC was injected subcutaneously. Tumors volume was evaluated every 4 days. Following 4 weeks, mice were killed, and tumors were weighted.

### Statistical analysis

Data were presented as mean ± SD of at least three independently conducted assays. Student’s t-test or one-way ANOVA was used for comparison of groups. Pearson Correlation Coefficient was applied for testing the association among SNHG17, CTNNB1 and miR-506-3p expressions. All statistical analyses were undertaken with SPSS 17.0 software (SPSS, Chicago, IL, USA). P < 0.05 was considered statistically significant.

## Results

### SNHG17 was highly expressed in glioma and promoted the process of glioma

**Fig. 1:**
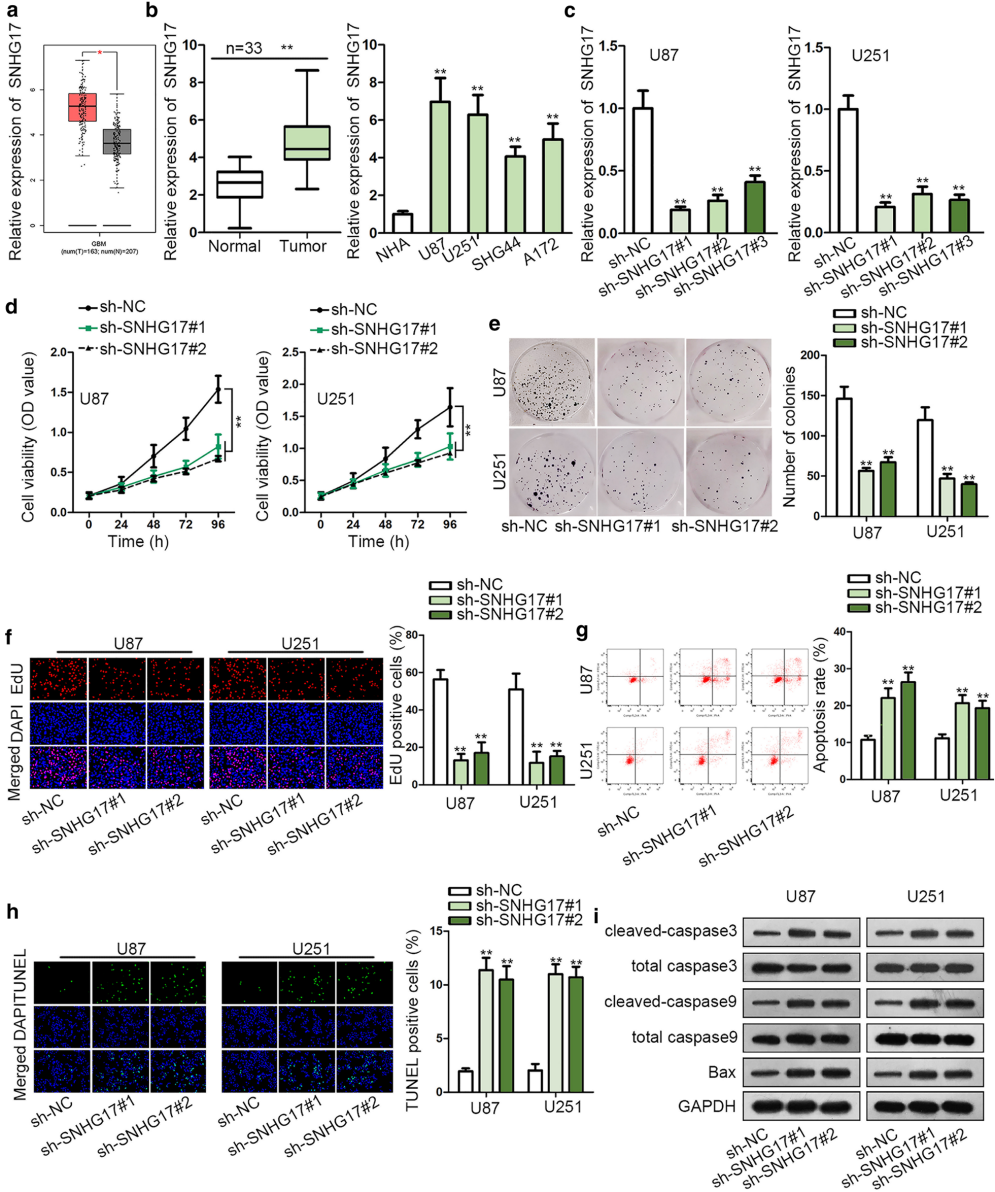
SNHG17 was highly expressed in glioma and promoted the process of glioma. **a** GEPIA database showed that SNHG17 expression was strongly expressed in glioma samples. **b** RT-qPCR displayed that SNHG17 expression was boosted in glioma tissues and cell lines. **c** RT-qPCR manifested SNHG17 was effectively knocked down in glioma cells. **d** CCK-8 assay revealed that cell viability was alleviated by SNHG17 suppression. **e** Colony formation assay uncovered the number of colonies was decreased as a result of SNHG17 depletion. **f** EdU assay unveiled that EdU positive cells declined due to SNHG17 silence. **g**, **h** Flow cytometry and TUNEL assays both unveiled that cell apoptosis was enhanced by down-regulation of SNHG17. **i** Western blot assay displayed that the protein level of cell apoptosis-related proteins (cleaved-caspase3, cleaved-caspase9 and Bax) was all increased attributed to SNHG17 deficiency. **P < 0.01

For investigating the role of SNHG17 in glioma, we searched GEPIA database (http://gepia.cancer-pku.cn/) and found SNHG17 expression was up-regulated in glioma samples (Fig. 1a). RT-qPCR showed that SNHG17 expression was boosted in glioma tissues and cell lines (Fig. 1b). These results implied that SNHG17 might participate in glioma. Thus we further explored the detailed function of SNHG17 in glioma. To begin with, SNHG17 was effectively knocked down in glioma cells (Fig. 1c). Sh-SNHG17#1 and sh-SNHG17#2 presented better knockdown efficiency and were selected in the following loss-of-function assays. Cell proliferative ability among glioma cells was detected in CCK-8, colony formation and EdU assays. According to CCK-8 assay outcome, cell viability was alleviated by SNHG17 suppression (Fig. 1d). Colony formation assay revealed the number of colonies was decreased as a result of SNHG17 depletion (Fig. 1e). Likewise, EdU positive cells were reduced due to SNHG17 silence (Fig. 1f). Furthermore, flow cytometry and TUNEL assay both unveiled that cell apoptosis was enhanced by down-regulation of SNHG17 (Fig. 1g, h). Western blot assay displayed that the protein level of cell apoptosis-related proteins (cleaved-caspase3, cleaved-caspase9 and Bax) were all increased attributed to SNHG17 deficiency (Fig. 1i). All in all, SNHG17 was highly expressed in glioma and down-regulation of SNHG17 blocked glioma progression.

### SNHG17 up-regulation was induced by YY1

**Fig. 2:**
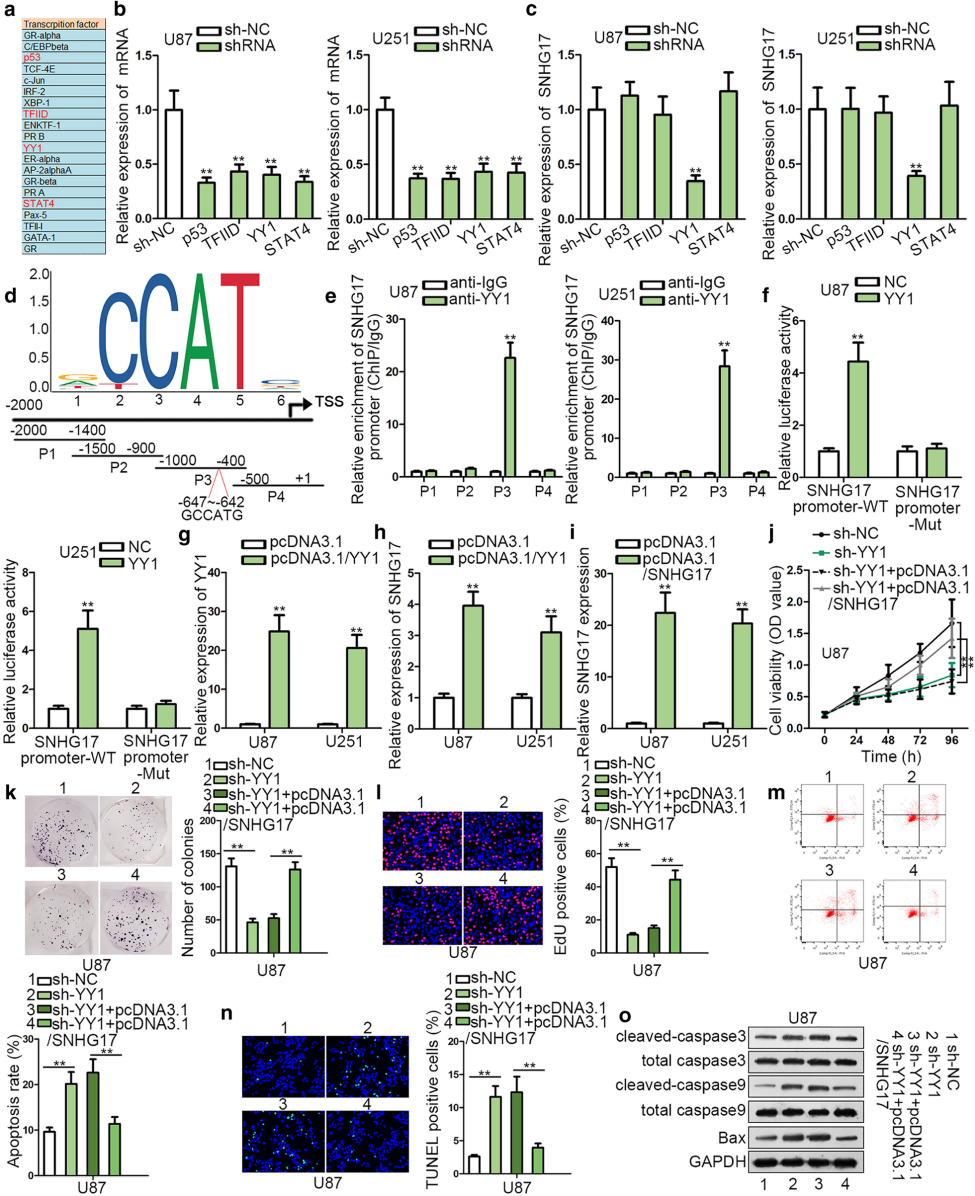
SNHG17 up-regulation was induced by YY1. **a** RT-qPCR demonstrated that p53, TFIID, YY1 and STAT4 expressions were elevated in glioma tissues among 20 transcription factors predicted to affect the transcription of SNHG17 by PRPOMO database. **b** RT-qPCR showed that the expression of p53, TFIID, YY1 and STAT4 was knocked down in glioma cells. **c** RT-qPCR manifested that the expression of SNHG17 was reduced by YY1 silence. **d** JASPAR database presented the binding motif of YY1 and the binding region of YY1 on SNHG17 promoter. **e** ChIP assay demonstrated that P3 region (− 647 to − 642, GCCATG) was responsive to YY1-mediated transcription of SHNG17. **f** Luciferase reporter assay illustrated that YY1 could bind with SNHG17 promoter. **g** RT-qPCR displayed the expression of YY1 in glioma cells. **h** RT-qPCR showed the expression of SNHG17. **i** RT-qPCR examined the SNHG17 expression. **j**–**l** CCK-8, colony formation and EdU assays revealed that cell proliferative capacity was hampered by YY1 depletion and was promoted by overexpression of SNHG17. **m**, **n** Flow cytometry and TUNEL assays unveiled that enhanced cell apoptosis by silence of YY1 was reversed by SNHG17 up-regulation. **o** Western blot assay demonstrated that increased protein level of cleaved-caspase3, cleaved-caspase9 and Bax by YY1 ablation was rescued by SNHG17 enhancement. **P < 0.01

To make clear how SNHG17 was highly expressed in glioma, the upstream of SNHG17 was probed. By use of PROMO database (http://alggen.lsi.upc.es/cgi-bin/promo_v3/promo/promoinit.cgi?dirDB=TF_8.3), 20 transcription factors were predicted to potentially bind to SNHG17 promoter, and their expressions in glioma cells were investigated. It turned out that p53, TFIID, YY1 and STAT4 expressions were elevated in glioma cells (Fig. 2a). Next, p53, TFIID, YY1 and STAT4 were knocked down in glioma cells (Fig. 2b). RT-qPCR manifested that the expression of SNHG17 was reduced by YY1 silence rather than p53, TFIID and STAT4 (Fig. 2c), suggesting that YY1 might be the upstream of SNHG17. According to JASPAR database (http://jaspar.genereg.net/), the binding motif of YY1 and YY1 binding site on SNHG17 promoter were shown in Fig. 2d. ChIP assay demonstrated P3 region which contained the YY1 site (− 647 to − 642, GCCATG) was responsive to YY1-mediated transcription of SNHG17 while no evident changes could be found in others (Fig. 2e). Luciferase reporter assay illustrated that the luciferase of SNHG17 promoter-WT was increased by YY1, but in the NC group and SNHG17 promoter-Mut group, the luciferase showed no change (Fig. 2f), indicating that YY1 could bind with SNHG17 promoter. Then, favorable overexpression efficiency of YY1 was obtained in glioma cells (Fig. 2g). By overexpression of YY1, the expression of SNHG17 was also enhanced (Fig. 2h). We analyzed relationship between SNHG17 and YY1 expression among 33 glioma tissues and found that YY1 was positively correlated with SNHG17 (Additional file 1: Figure S1A). Thereafter, SNHG17 was overexpressed by pcDNA3.1/SNHG17 vector, which resulted in a striking increase of SNHG17 expression (Fig. 2i). We carried out functional assays to verify the effects of up-regulation of SNHG17 on glioma progression. Outcomes of colony formation revealed that proliferative abilities were increased by up-regulation of SNHG17 while apoptosis examined via flow cytometry appeared no distinct changes due to the augment of SNHG17 (Additional file 1: Figure S1B, C). Subsequently, we performed rescue assays to illustrate how YY1 regulated the process of glioma by modulating SNHG17 expression. First, we confirmed that transfection of sh-YY1 significantly reduced YY1 mRNA and protein expression, but co-transfection of SNHG17 triggered no significant change in the expression of YY1 at both mRNA and protein levels (Additional file 2: Figure S2A). Also, the level of SNHG17 was reduced by sh-YY1, and overexpression of SNHG17 recovered SNHG17 level in U87 cells (Additional file 2: Figure S2A). These results further indicated that SNHG17 was a downstream target for YY1. Cell proliferation and colony generation were hampered by YY1 depletion and then such effect was neutralized by overexpression of SNHG17 (Fig. 2j–l). In addition, enhanced cell apoptosis by silence of YY1 was reversed by SNHG17 up-regulation (Fig. 2M, N). Increased proteins levels of cleaved-caspase3, cleaved-caspase9 and Bax caused by YY1 down-regulation were rescued via SNHG17 enhancement (Fig. 2o). In summary, YY1 could promote SNHG17 transcription and up-regulate its expression. Furthermore, YY1 could accelerate the growth of glioma via up-regulating SNHG17 expression.

### SNHG17 activated Wnt/β-catenin signaling pathway in glioma

**Fig. 3:**
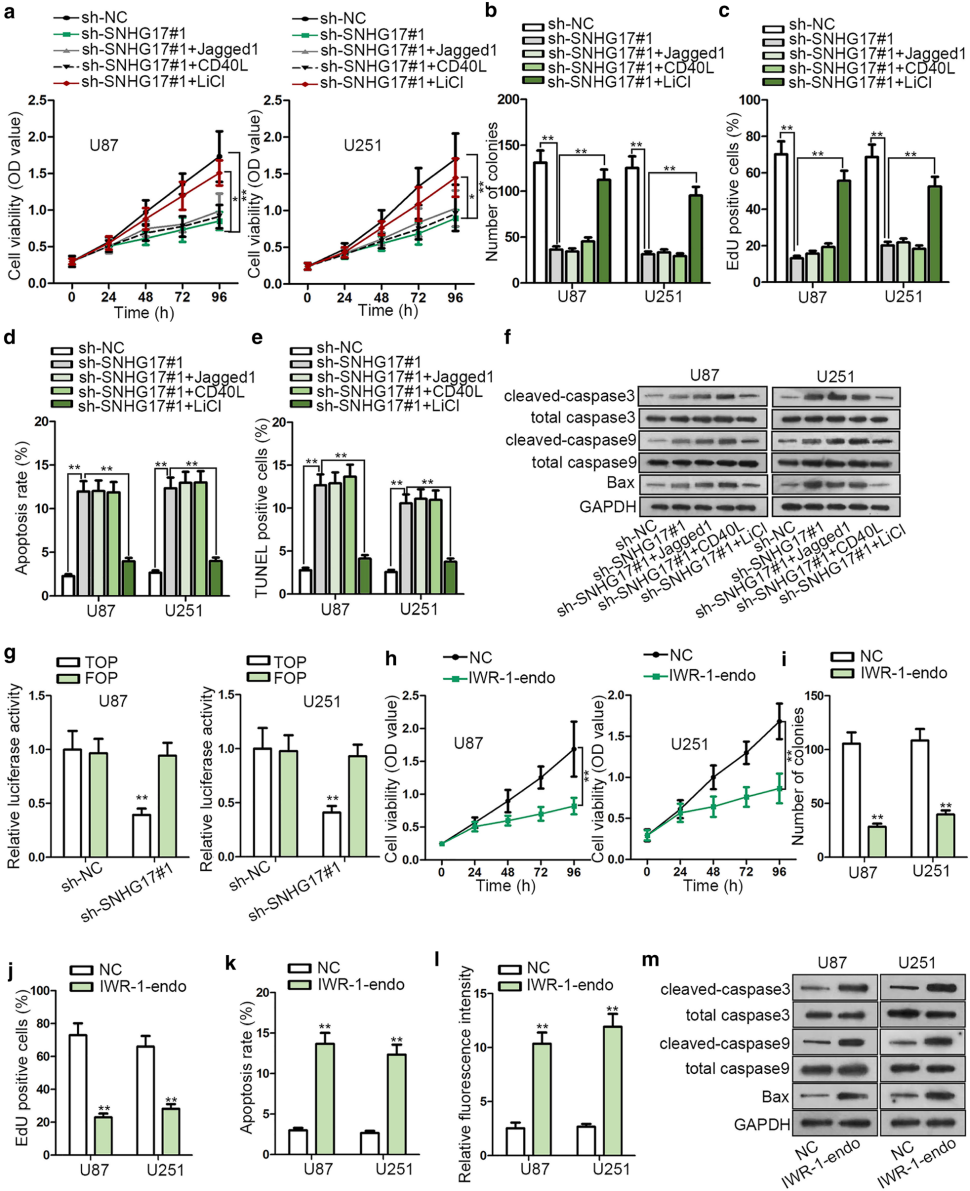
SNHG17 activated Wnt/β-catenin signaling pathway in glioma. **a** CCK-8 assay demonstrated that by adding the activator of Wnt/β-catenin signaling pathway (LiCl), the suppressed function of SNHG17 depletion on cell proliferation was rescued. Jagged1 and CD40L were added to rescue the effects of SNHG17 knockdown as well. **b**, **c** Colony formation and EdU assay manifested that the cell proliferation ability repressed by SNHG17 silence was only recovered by adding LiCl rather than Jagged1 and CD40L. **d**, **e** Flow cytometry and TUNEL assays manifested that cell apoptosis induced by SNHG17 down-regulation was abolished by adding LiCl rather than Jagged1 and CD40L. **f** Western blot assay uncovered the protein level of cleaved-caspase3, cleaved-caspase9 and Bax. **g** TOP/FOP flash assay showed the luciferase activity of TOP and FOP vectors. **h**–**j** CCK-8, colony formation and EdU assays unveiled that cell proliferation was blocked by adding the inhibitor of Wnt/β-catenin signaling pathway. **k**, **l** Flow cytometry and TUNEL assays demonstrated that cell apoptosis was enhanced via adding the inhibitor of Wnt/β-catenin signaling pathway. **m** The protein level of cleaved-caspase3, cleaved-caspase9 and Bax was elevated in western blot assay. *P < 0.05, **P < 0.01

It was reported that various signaling pathways were implicated with cancer progression, therefore we were curious about whether there was a signaling pathway involved in glioma. Jagged-1 was used as acticator in Notch signaling pathway [17]. CD40L was the main part of CD40/CD40L pathway [18]. LiCl was the activator of Wnt/β-catenin signaling pathway [19]. Afterwards, the activators of the three typical pathways which were more widely proposed to exert function in cancers were respectively added in glioma cells. CCK-8 assay displayed that by adding LiCl, the suppressed function of SNHG17 depletion on cell proliferation was rescued (Fig. 3a). Consistently, colony formation and EdU assay manifested that the repressed cell proliferation ability under SNHG17 silence was only recovered by adding LiCl (Fig. 3b, c). The inducing effect of SNHG17 down-regulation on cell apoptosis was abolished through adding LiCl (Fig. 3d, e). The protein level of cleaved-caspase3, cleaved-caspase9 and Bax was enhanced by SNHG17 ablation, but adding LiCl counteracted this effect (Fig. 3f). TOP/FOP flash assay showed that the luciferase activity of TOP vector was hindered by SNHG17 suppression, while the luciferase activity of FOP vector displayed no significant change (Fig. 3g). All the data suggested that SNHG17 activated Wnt/β-catenin signaling pathway in glioma. Furthermore, the function of Wnt/β-catenin signaling pathway in glioma was detected. We used IWR-1-endo to detect the influence of Wnt signaling pathway on glioma progression. Cell proliferation was curbed by adding IWR-1-endo (Fig. 3h–j). Cell apoptosis was promoted due to adding IWR-1-endo (Fig. 3k, l). The protein level of cleaved-caspase3, cleaved-caspase9 and Bax was elevated because of adding IWR-1-endo (Fig. 3m). In conclusion, SNHG17 activated Wnt/β-catenin signaling pathway to aggravate the course of glioma.

### SNHG17 up-regulated the expression of CTNNB1 by sponging miR-506-3p

**Fig. 4:**
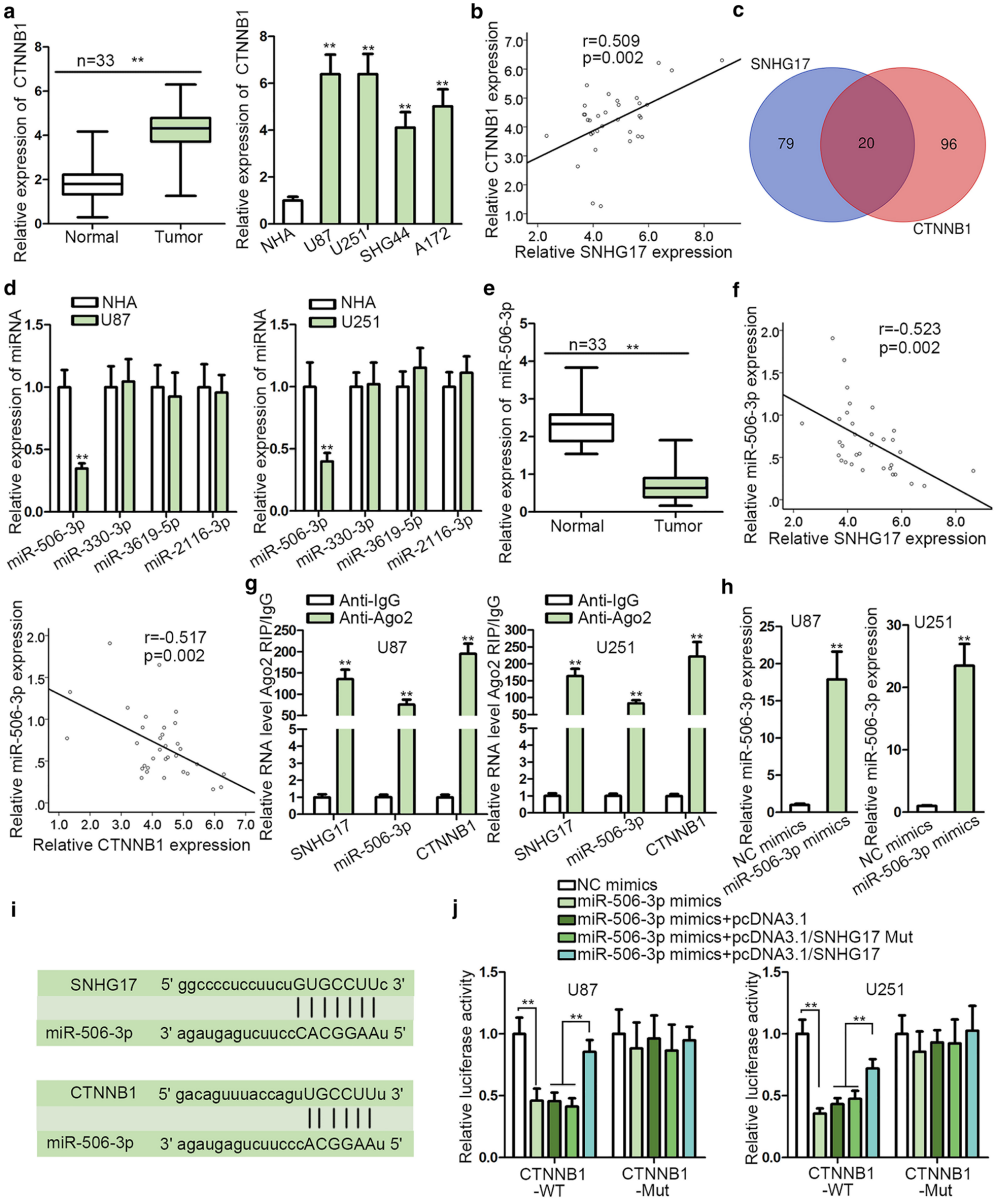
SNHG17 up-regulated the expression of CTNNB1 by sponging miR-506-3p. **a** CTNNB1 expression was tested to be up-regulated in glioma tissues and cell lines. **b** Pearson’s correlation analysis demonstrated that CTNNB1 expression had a positive correlation with SNHG16 expression. **c** StarBase database uncovered 20 putative miRNAs was predicted to not only be sponged by SNHG17 but also targeted CTNNB1. **d** RT-qPCR exhibited the expressions of miR-506-3p, miR-330-3p, miR-3619-5p and miR-2116-3p in glioma cells. **e** RT-qPCR unveiled that the expression of miR-506-3p was down-regulated in glioma tissues. **f** Pearson’s correlation analysis illustrated that miR-506-3p expression was negatively associated with SNHG17 and CTNNB1. **g** RIP assay displayed that SNHG17, miR-506-3p and CTNNB1 were together enriched in RISC complex. **h** RT-qPCR showed that miR-506-3p was overexpressed by miR-506-3p mimics. **i** The binding site for SNHG17 and miR-506-3p as well as the binding site for miR-506-3p and CTNNB1 were exhibited (**j**) Luciferase reporter assay illustrated that SNHG17 competitively bound with miR-506-3p to up-regulate CTNNB1. **P < 0.01

SNHG17 activated Wnt/β-catenin signaling pathway was aforementioned, but how SNHG17 activated Wnt/β-catenin signaling pathway remained to be elucidated. Given that CTNNB1 was the mRNA of β-catenin; we speculated SNHG17 might regulate Wnt/β-catenin signaling pathway via up-regulating the expression of CTNNB1 in glioma. Via RT-qPCR, up-regulated expression of CTNNB1 was observed in glioma tissues and cell lines (Fig. 4a). Pearson’s correlation analysis demonstrated that CTNNB1 expression had a positive correlation with SNHG16 expression (Fig. 4b). Based on starBase database (http://starbase.sysu.edu.cn/), 20 putative miRNAs were predicted to not only be sponged by SNHG17 but also targeted CTNNB1 (Fig. 4c and Additional file 1: Figure S1D). Among them, miR-506-3p, miR-330-3p, miR-3619-5p and miR-2116-3p had never been explored in glioma but they had been reported to play anti-tumor role in other cancers. Hence, they had high possibility to play the same role in glioma. To validate this assumption, we conducted the RT-qPCR assays and found that only miR-506-3p, miR-330-3p, miR-3619-5p and miR-2116-3p were up-regulated by SNHG17 knockdown in glioma cells (Additional file 1: Figure S1E). RT-qPCR showed only miR-506-3p expression was silenced in glioma cells versus normal NHA cells (Fig. 4d). Similarly, miR-506-3p expression was down-regulated in glioma tissues (Fig. 4e). Pearson’s correlation analysis illustrated that miR-506-3p expression was negatively associated with SNHG17 and CTNNB1 expression (Fig. 4f). The results of RNA pull down manifested that SNHG17 and CTNNB1 could be pulled down by biotinylated miR-506-3p probe (Additional file 1: Figure S1F–G), which meant miR-506-3p could bind to both SNHG17 and CTNNB1. Afterwards, the outcome of RIP assay displayed that SNHG17, miR-506-3p and CTNNB1 were together enriched in the precipitates of Ago2 antibody but not of IgG antibody, which suggested that they coexisted in RISC complex (Fig. 4g). Subsequently, miR-506-3p mimics were transfected into cells and we found that transfection of miR-506-3p mimics resulted in an evident up-regulation of miR-506-3p expression (Fig. 4h). In Fig. 4i, the binding site between SNHG17 and miR-506-3p as well as the binding site between miR-506-3p and CTNNB1 was exhibited. The data of luciferase reporter assay manifested that the luciferase activity of CTNNB1-WT depressed by miR-506-3p up-regulation was restored by overexpression of wild SNHG17 but not mutant SNHG17 overexpression (Fig. 4j), implying that SNHG17 competitively bound with miR-506-3p to up-regulate the expression of CTNNB1. In addition, we confirmed that the levels of CTNNB1 and its protein β-catenin were downregulated by miR-506-3p, and the overexpression of SNHG17, rather than SNHG17 Mut (with the miR-506-3p site mutated), can restore the levels of CTNNB1 and β-catenin (Additional file 1: Figure S1H). On the whole, SNHG17 elevated the expression of CTNNB1 by sponging miR-506-3p.

### Overexpression of CTNNB1 rescued the SNHG17 depletion-mediated inhibition on the progression of glioma

**Fig. 5:**
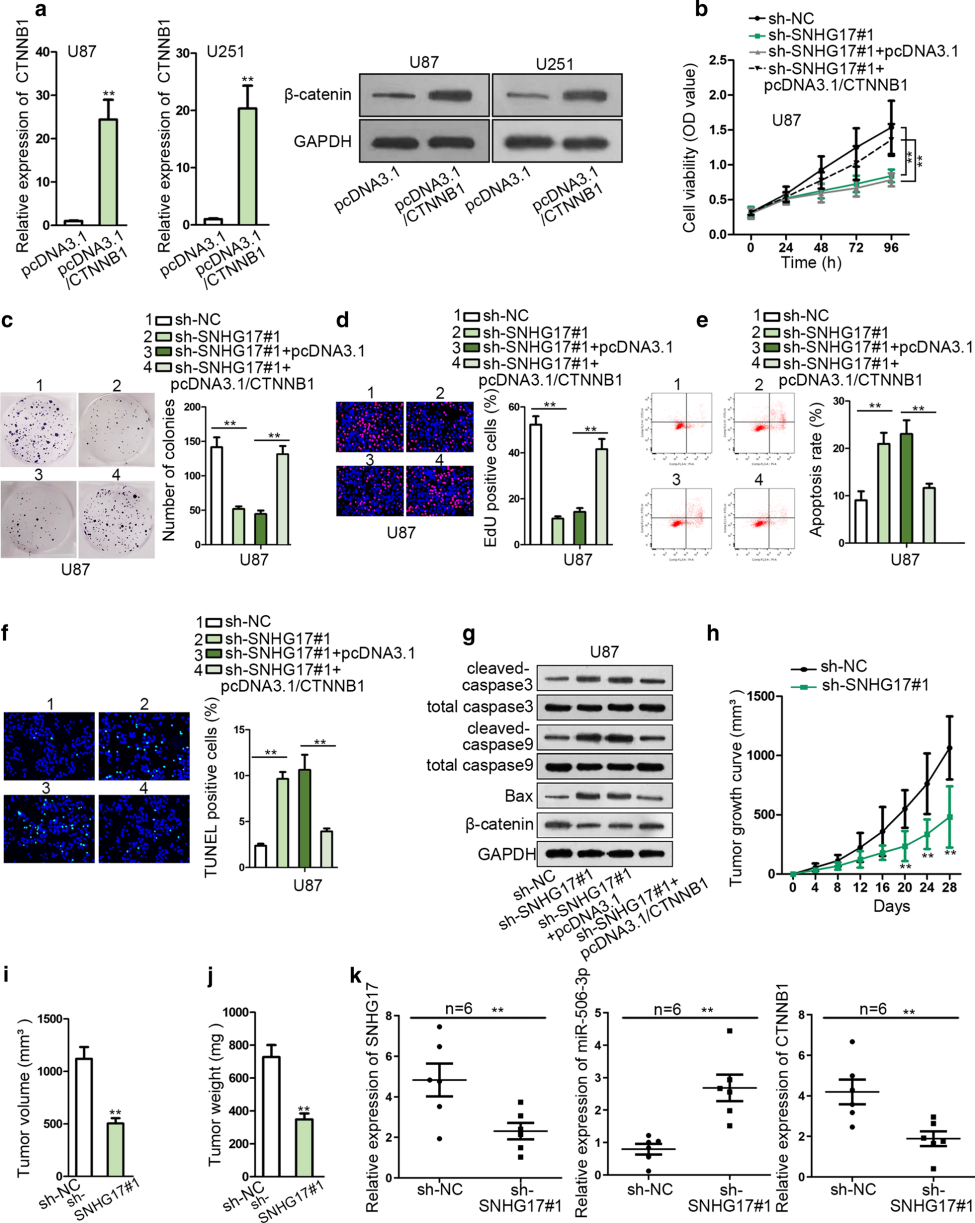
Overexpression of CTNNB1 rescued the SNHG17 depletion mediated inhibition on the progression of glioma. **a** RT-qPCR manifested that CTNNB1 was effectively up-regulated in glioma cells. **b**–**d** The inhibitory effect on cell proliferation caused by SNHG17 silence was countervailed by overexpression of CTNNB1. **e**, **f** Flow cytometry and TUNEL assays illustrated that the up-regulation of CTNNB1 abrogated the suppression of SNHG17 induced increase of cell apoptosis in U87 cells. **g** Western blot assay corroborated that attenuation of SNHG17 promoted the protein expression of cleaved-caspase3, cleaved-caspase9 and Bax, while overexpression of CTNNB1 reversed this effect. **h** The tumor growth curve indicted tumor size was suppressed by ablation of SNHG17 in vivo. **i**, **j** Tumor volume and tumor weight were both blocked as a result of SNHG17 knockdown. **k** RT-qPCR showed SNHG17 and CTNNB1 expression was reduced while miR-506-3p expression was induced by SNHG17 knockdown in glioma tissues. **P < 0.01

Finally, a series of rescue experiments were performed in U87 cells. Prior to all, levels of CTNNB1 and its protein β-catenin were effectively up-regulated in glioma cells after the transfection of pcDNA3.1/CTNNB1 (Fig. 5a). From the data of CCK-8, colony formation and EdU assays, we observed that the inhibitory effect of SNHG17 silence on cell proliferation was countervailed by overexpression of CTNNB1 (Fig. 5b–d). Besides, up-regulation of CTNNB1 abrogated the increase of cell apoptosis caused by the SNHG17 knockdown in U87 cells (Fig. 5e, f). Attenuation of SNHG17 promoted the protein expression of cleaved-caspase3, cleaved-caspase9 and Bax and reduced the expression of β-catenin, while overexpression of CTNNB1 reversed this effect (Fig. 5g). To sum up, overexpression of CTNNB1 rescued the SNHG17 depletion-mediated inhibition on the growth of glioma.

Furthermore, the role of SNHG17 in vivo was investigated. The tumor growth curve indicted that tumor generation was retarded by ablation of SNHG17 in vivo (Fig. 5h). Consistently, tumor volume and tumor weight were both reduced as a result of SNHG17 knockdown (Fig. 5i, j). All the experimental data from in vivo assay illustrated that SNHG17 promoted tumor growth in vivo. The expressions of SNHG17, miR-506-3p and CTNNB1 in tissues were detected by RT-qPCR, showing that SNHG17 and CTNNB1 expressions were reduced but miR-506-3p expression was increased in glioma tissues with SNHG17 overexpression compared with sh-NC group (Fig. 5k). In general, SNHG17 promoted glioma progression through up-regulating CTNNB1 expression.

## Discussion

Glioma is a ubiquitous type of cancer possessing high morbidity and mortality [20]. LncRNAs are widely acknowledged as novel biomarkers for glioma treatment. For example, lncRNA NEAT1 drove the development of glioma through inhibiting miR-132 to enhance the expression of SOX2 [21]. LncRNA PVT1 promoted glioma tumorigenesis and progression by targeting miR-128-3p/GREM1 axis and regulating BMP signaling pathway [22]. LncRNA MALAT1 was reported to have malignant status and predicted poor prognosis in glioma [23]. LncRNA SNHG17 was validated to promote the progression of NSCLC and gastric cancer [24, 25]. In present study, SNHG17 expression was identified to be elevated in glioma tissues and cell lines. Inhibition of SNHG17 blocked cell proliferation and accelerated cell apoptosis in glioma cells. Moreover, SNHG17 triggered tumor growth in vivo. Besides, overexpression of SNHG17 contributed to proliferation in glioma. All the data indicated that SNHG17 acted as a tumor promoter in glioma.

Transcription factors are implicated with cancer development [26]. Transcription factors can bind with lncRNAs promoter to increase their expression [27, 28]. Transcription factor YY1 was found to regulate lncRNA expression in colorectal cancer [29], but whether it regulated SNHG17 in glioma was still unclear. In our study, YY1 was corroborated to bind with SNHG17 promoter and enhanced its expression. Besides, YY1 also facilitated the progression of glioma. All the data implied that YY1 increased the expression of SNHG17 through binding with SNHG17 promoter to boost the development of glioma.

Wnt/β-catenin signaling pathway was confirmed to play a central role in multiple cellular activities [30, 31]. In present study, we found that LiCl, rather than Jagged1 and CD40L, was the activator to restore the effects of SNHG17 down-regulation on glioma. Thus, we assumed that SNHG17 could regulate the activity of Wnt/β-catenin signaling pathway. In addition, by adding the inhibitor of Wnt/β-catenin signaling pathway (IWR-1-endo), cell proliferation was suppressed and cell apoptosis was enhanced among glioma cells. All the data suggested that SNHG17 activated Wnt/β-catenin signaling pathway to promote the progression of glioma.

To further investigate why SNHG17 activated Wnt/β-catenin signaling pathway in glioma, we hypothesized that SNHG17 might influence CTNNB1 expression to activate Wnt/β-catenin signaling pathway. CTNNB1, the mRNA which encoded β-catenin, was up-regulated in tissues and cells of glioma. We showed that CTNNB1 was positively correlated with SNHG17. Given that lncRNAs regulated mRNA expression by sponging miRNAs, we explored whether SNHG17 affected CTNNB1 expression in this pattern. We used bioinformatics website to select out 20 miRNAs that could both bind to SNHG17 and CTNNB1. Furthermore, we verified that SNHG17 negatively regulated miR-506-3p expression meanwhile miR-506-3p negatively regulated CTNNB1 expression in glioma. The luciferase reporter assay and RIP assay certified the affinity among miR-506-3p with SNHG17 and CTNNB1. Rescue experiments illustrated that overexpression of CTNNB1 offset the inhibitory effect resulted from SNHG17 depletion in glioma cells.

## Conclusion

SNHG17 induced by YY1 facilitated the glioma progression through targeting miR-506-3p/CTNNB1 axis to activate Wnt/β-catenin signaling pathway. These findings might provide a novel prospect for the diagnosis and therapy for glioma.

## Supplementary information

**Additional file 1: Figure S1.** (A) Pearson’s correlation analysis demonstrated that YY1 was positively corrected with SNHG17. (B-C) The effects of SNHG17 overexpression on proliferation and apoptosis were shown. (D) 20 miRNAs could bind to both CTNNB1 and SNHG17. (E) RT-qPCR evaluated 20 miRNAs expressions in cells transfected with sh-SNHG17#1. (F-G) RNA pull down was used to validate the interactions of miR-506-3p with SNHG17 and CTNNB1. (H) RT-qPCR and western blot were conducted to measure CTNNB1 expression and the level of its protein β-catenin in cells transfected with NC mimics, miR-506-3p mimics, miR-506-3p mimics + pcDNA3.1, miR-506-3p mimics + pcDNA3.1/SNHG17 (Mut) or miR-506-3p mimics + pcDNA3.1/SNHG17. ^**^P < 0.01.

**Additional file 2: Figure S2.** (A) RT-qPCR results of YY1 and SNHG17 and western blot result of YY1 in U87 cells transfected with sh-NC, sh-YY1, or sh-YY1 + pcDNA3.1/SNHG17. ^**^P < 0.01.

## Data Availability

Research data are not shared.

## Abbreviations

lncRNAs: long noncoding RNAs
ATCC: American Type Culture Collection
DMEM: Dulbecco’s Modified Eagle Medium
cDNA: complementary DNA
CCK-8: Cell Counting Kit 8
ECL: enhanced chemiluminescence substrate system
ChIP: chromatin immunoprecipitation
RIP: RNA immunoprecipitation
