# Supplementary figures and images for: Long noncoding RNA SNHG17 induced by YY1 facilitates the glioma progression through targeting miR-506-3p/CTNNB1 axis to activate Wnt/β-catenin signaling pathway
Source: Cancer Cell Int. 2020 Jan 28;20:29. doi: 10.1186/s12935-019-1088-3 (PMC6988207; doi:10.1186/s12935-019-1088-3)

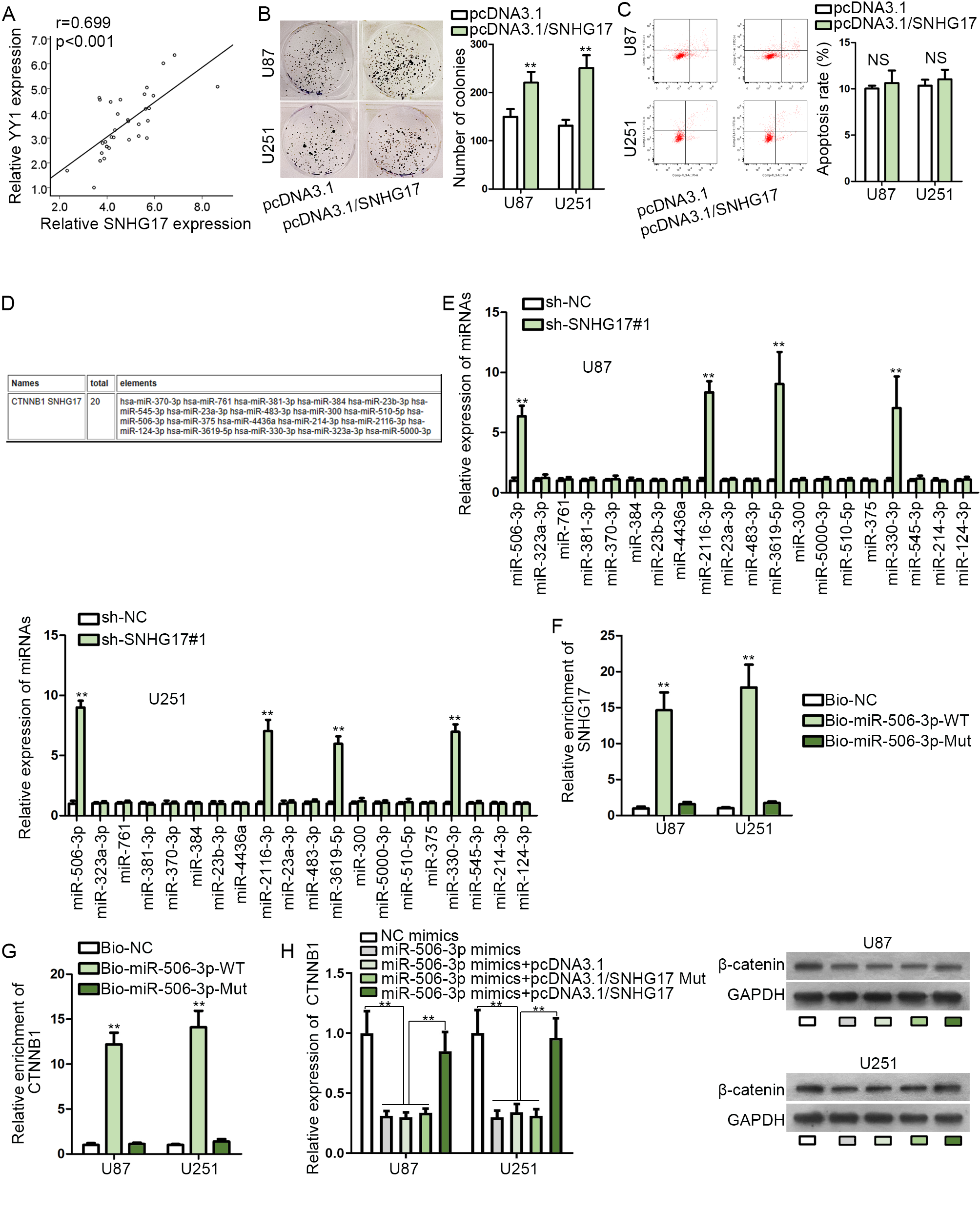

Supplement: Supplementary file 1 — Additional file 1: Figure S1. (A) Pearson’s correlation analysis demonstrated that YY1 was positively corrected with SNHG17. (B-C) The effects of SNHG17 overexpression on proliferation and apoptosis were shown. (D) 20 miRNAs could bind to both CTNNB1 and SNHG17. (E) RT-qPCR evaluated 20 miRNAs expressions in cells transfected with sh-SNHG17#1. (F-G) RNA pull down was used to validate the interactions of miR-506-3p with SNHG17 and CTNNB1. (H) RT-qPCR and western blot were conducted to measure CTNNB1 expression and the level of its protein β-catenin in cells transfected with NC mimics, miR-506-3p mimics, miR-506-3p mimics + pcDNA3.1, miR-506-3p mimics + pcDNA3.1/SNHG17 (Mut) or miR-506-3p mimics + pcDNA3.1/SNHG17. **P < 0.01. [file 12935_2019_1088_MOESM1_ESM.tif]

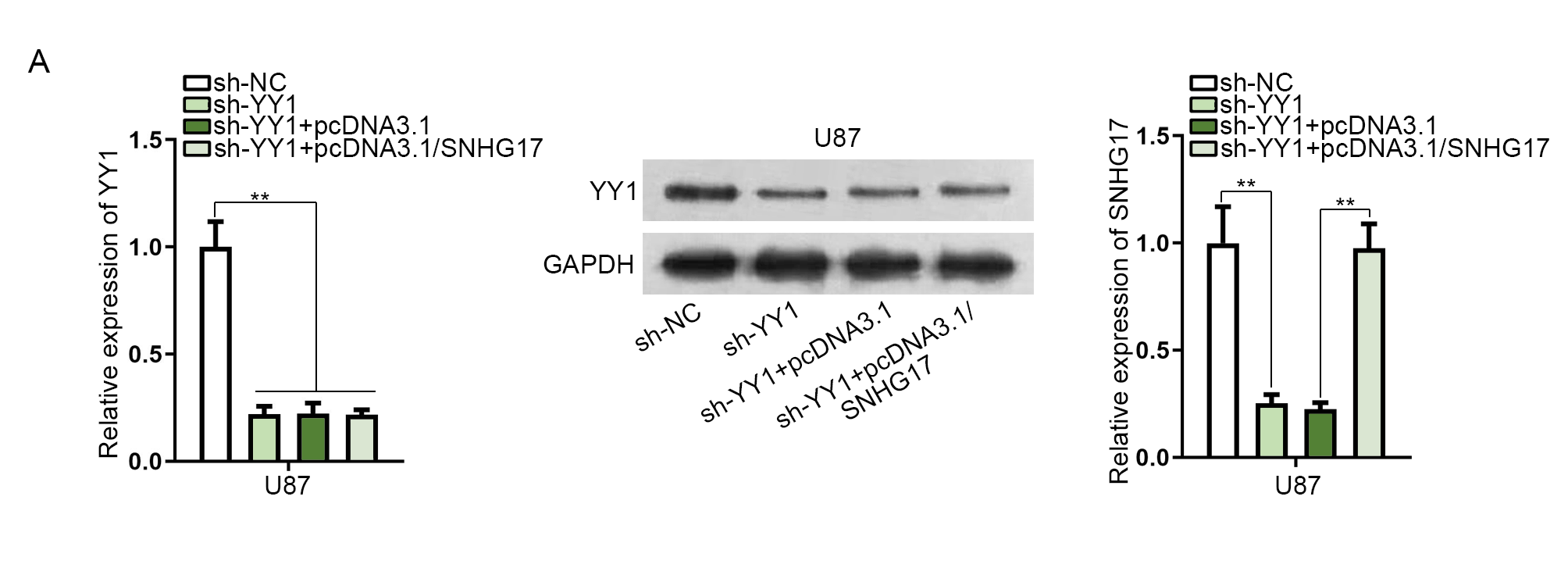

Supplement: Supplementary file 2 — Additional file 2: Figure S2. (A) RT-qPCR results of YY1 and SNHG17 and western blot result of YY1 in U87 cells transfected with sh-NC, sh-YY1, or sh-YY1 + pcDNA3.1/SNHG17. **P < 0.01. [file 12935_2019_1088_MOESM2_ESM.tif]
